# A bioinformatics approach reveals novel interactions of the OVOL transcription factors in the regulation of epithelial – mesenchymal cell reprogramming and cancer progression

**DOI:** 10.1186/1752-0509-8-29

**Published:** 2014-03-10

**Authors:** Hernan Roca, Manjusha Pande, Jeffrey S Huo, James Hernandez, James D Cavalcoli, Kenneth J Pienta, Richard C McEachin

**Affiliations:** 1 Department of Periodontics and Oral Medicine, University of Michigan, Ann Arbor, MI, USA; 2 Department of Computational Medicine and Bioinformatics, University of Michigan, Ann Arbor, MI, USA; 3 Oncology Center, Pediatric Oncology, The Johns Hopkins University, Baltimore, MD, USA; 4 The Brady Urological Institute and Department of Urology, Johns Hopkins Medical Institutions, Baltimore, MD, USA

**Keywords:** Metastasis, Migration, Tumor progression, Systems biology, Transcription factors, Signal transduction, Therapeutics

## Abstract

**Background:**

Mesenchymal to Epithelial Transition (MET) plasticity is critical to cancer progression, and we recently showed that the OVOL transcription factors (TFs) are critical regulators of MET. Results of that work also posed the hypothesis that the OVOLs impact MET in a range of cancers. We now test this hypothesis by developing a model, OVOL Induced MET (OI-MET), and sub-model (OI-MET-TF), to characterize differential gene expression in MET common to prostate cancer (PC) and breast cancer (BC).

**Results:**

In the OI-MET model, we identified 739 genes differentially expressed in both the PC and BC models. For this gene set, we found significant enrichment of annotation for BC, PC, cancer, and MET, as well as regulation of gene expression by AP1, STAT1, STAT3, and NFKB1. Focusing on the target genes for these four TFs plus the OVOLs, we produced the OI-MET-TF sub-model, which shows even greater enrichment for these annotations, plus significant evidence of cooperation among these five TFs. Based on known gene/drug interactions, we prioritized targets in the OI-MET-TF network for follow-on analysis, emphasizing the clinical relevance of this work. Reflecting these results back to the OI-MET model, we found that binding motifs for the TF pair AP1/MYC are more frequent than expected and that the AP1/MYC pair is significantly enriched in binding in cancer models, relative to non-cancer models, in these promoters. This effect is seen in both MET models (solid tumors) and in non-MET models (leukemia). These results are consistent with our hypothesis that the OVOLs impact cancer susceptibility by regulating MET, and extend the hypothesis to include mechanisms not specific to MET.

**Conclusions:**

We find significant evidence of the OVOL, AP1, STAT1, STAT3, and NFKB1 TFs having important roles in MET, and more broadly in cancer. We prioritize known gene/drug targets for follow-up in the clinic, and we show that the AP1/MYC TF pair is a strong candidate for intervention.

## Background

Cancer progression is characterized, in part, by altered or aberrant transcription factor (TF) function, leading to changes in expression of cancer related genes [1]. Mesenchymal to Epithelial Transition (MET) and its mirror process (Epithelial to Mesenchymal Transition, EMT) are critical to metastasis in cancer progression [2]. We recently demonstrated [3] a novel function of the OVOL1 (ovo-like 1, Entrez GeneID 5017) and OVOL2 (ovo-like 2, GeneID 58495) TFs as critical inducers of MET in prostate cancer. (Note that there is a human OVOL3 gene, GeneID 728361, but it is “provisional” and largely un-annotated so we excluded it from this analysis.) One of the outcomes of this recent work suggests the hypothesis that the OVOLs have roles in regulating MET in multiple cancers. This hypothesis is also consistent with our earlier work [4-8], where we found common underlying genetic etiology for related disease phenotypes. We also found in earlier work [6,7,9] that exploring this common underlying genetic etiology using a systems biology approach can lead to improved understanding of the related phenotypes and interactions among the genetic influences on them, and may point out potential clinically significant biomarkers or drug targets.

**Figure 1:**
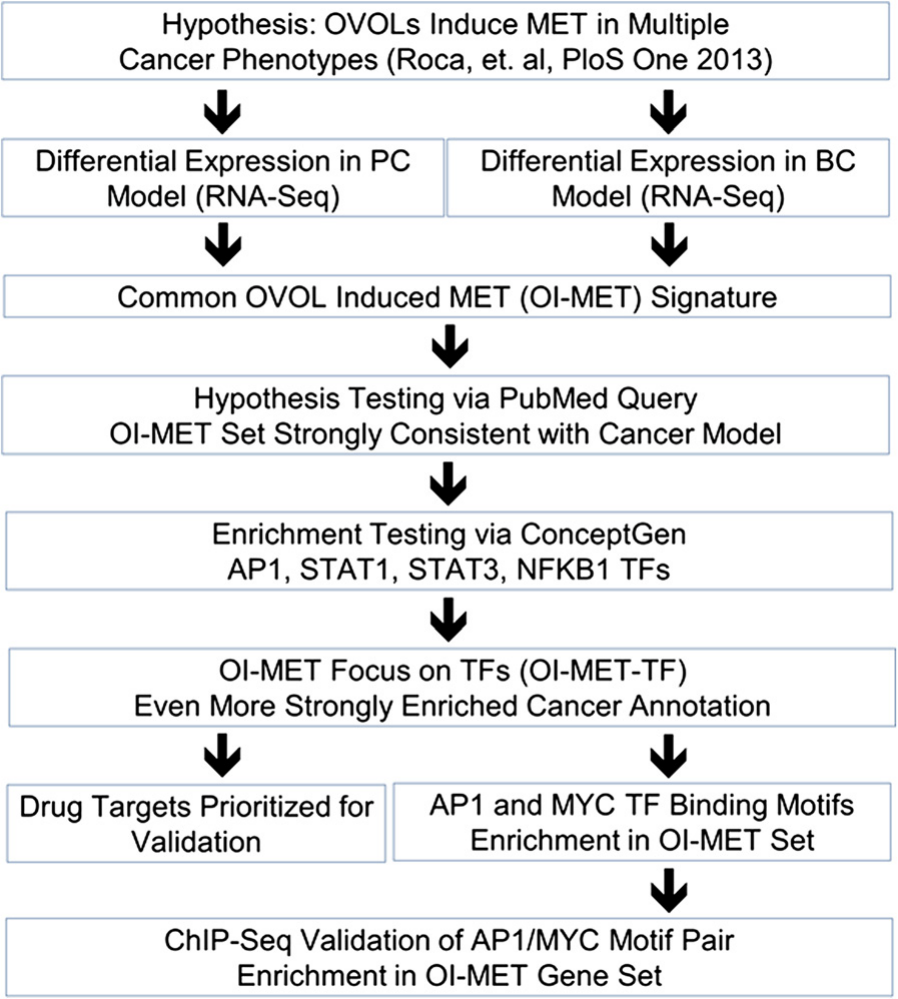
**Analysis flow.** We began the analysis with the hypothesis that the OVOLs impact MET in multiple cancers. We used RNA-Seq to identify sets of genes that are differentially expressed in response to OVOL TFs overexpression in BC and PC models. At the intersection of these sets are genes that are differentially expressed in OVOL Induced MET (OI-MET) across these two cancer models. We test the hypothesis that this set should be enriched for genes annotated for association with cancer, breast cancer, prostate cancer, and MET. We find annotation consistent with this hypothesis, as well as annotation for regulation of gene expression by AP1, STAT1, STAT3, and NFKB1 TFs. Pursuing this secondary hypothesis, we developed the OI-MET-TF model, based on the genes annotated as being regulated by these TFs and the OVOL TFs. Genes in the OI-MET-TF network are even more significantly enriched for cancer, breast cancer, prostate cancer, and MET annotation than the OI-MET set. Within the OI-MET-TF set, we identified genes documented to be drug targets and prioritized them for validation and near-term clinical follow up. Reflecting our inference from the OI-MET-TF model back to the OI-MET model, we found enrichment of AP1/MYC binding motif pairs in the promoters of the OI-MET gene set, suggesting the hypothesis that the AP1/MYC TF pair is important in regulating this gene set. Testing this hypothesis based on ChIP-Seq data, we find significant evidence consistent with this hypothesis.

In the present work (Figure 1), we explore the hypothesis that the OVOL TFs induce MET (OI-MET) in multiple cancers, focusing on commonalities between prostate cancer (PC) and breast cancer (BC) models. We generate a common OI-MET gene expression signature, consistent with a common underlying genetic etiology for MET in PC and BC, and show that the OI-MET gene set is significantly enriched for cancer, BC, PC, and MET-associated genes. Using a systems biology approach, we identify regulation of gene expression as the primary influence of the OVOLs on MET in these two models, though this effect is indirect and depends on interaction with AP1, STAT1, STAT3, and NFKB1 TFs. We create an OI-MET-TF sub-model of the genes annotated as being regulated by the OVOLs and these other four TFs. We test this model for consistency with known genetic influences on MET, BC, PC and cancer, and find that there is significant evidence supporting the use of this network as a model of gene expression influences on MET, as well as BC and PC, and more generally in cancer. We reflect the inference from the OI-MET-TF model back to the larger set of all OI-MET genes and show that the effects of the OVOLs and the other TFs in the OI-MET-TF model are likely to be consistent in the larger set, with experimental data significantly in support of this hypothesis. In particular, we find significant evidence that the AP1/MYC TF pair has an important role in regulating gene expression in MET related to BC, PC, and to cancer in general.

## Results

### OI-MET gene expression signature

We established MET gene expression signatures to characterize changes of gene expression in models of PC and BC (Additional file 1). Previously we demonstrated a role for the OVOL-TFs in the induction of MET [3]. Furthermore, based on Oncomine [10] data, we found that the gene expression analysis of the OVOL-TFs significantly correlates with the expression of MET markers in multiple cancer types. We approached the discovery of the MET signature by over-expression of OVOL1, OVOL2, or both OVOLs in the mesenchymal prostate cancer (PC3-EMT14) and breast cancer (MDA-MB-231) cell lines. In the prostate cancer model, we analyzed the following cell lines: PC3-EMT14-OVOL1 (OVOL1 overexpression), PC3-EMT14-OVOL2 (OVOL2 overexpression) and PC3-Epi (epithelial cells that express OVOL1 and OVOL2 and from which the mesenchymal PC3-EMT14 were initially obtained) [3]. Each of these OVOL-expressing cell lines demonstrated a stable transition to the MET phenotype. We also confirmed the MET phenotype in these models by showing differential expression of critical MET markers including: up-regulation of E-Cad expression plus down-regulation of both vimentin and the EMT-inducing TF ZEB15 [3]. We performed a parallel analysis for BC using breast cancer MDA-MB-231 cells, a poorly differentiated mesenchymal-type [3]. In this model we analyzed the following cell lines: MDA-MB-231-OVOL1 (OVOL1 overexpression), MDA-MB-231-OVOL2 (OVOL2 overexpression) and MDA-MB-231- OVOL1/2 (overexpression of both OVOL1 and OVOL2 in MDA-MB-231 cells) [3]. As in the PC experiment, the BC OVOL-expressing cell lines demonstrated a stable transition to the MET phenotype and appropriate expression of MET and EMT related genes.

Given these two models of related cancer phenotypes, and testing our hypothesis that they should share underlying genetic influences, we searched for a common OI-MET gene expression signature for these two models. We assessed differential gene expression in each of the models of OI-MET by RNA-Seq, and established a set of genes representing the OI-MET expression signature for each model. In each model (BC and PC), we selected the union of sets of genes responsive to OVOL1, OVOL2, or both, using FDR ≤ 0.05 AND Fold Change ≥ ± 2.0 AND “test OK” thresholds (Additional file 1). This selection yielded 1,622 genes in BC and 2,692 genes in PC. Focusing on common underlying genetic etiology for these related phenotypes, the 739 genes at the intersection of these two sets (Additional file 1, MET signature 739 genes) represent a common OI-MET gene expression signature for these two cancer models. Of these 739 genes, 66% follow the same pattern (both up, or both down) in the comparison of OVOL1&2 treated cells across the BC and PC models. We included all 739 genes in this analysis, ignoring direction, to include all genes that show strong responses in both models and to minimize the assumptions required in gene selection. For responsive genes, the direction of expression change is frequently a result of transient factors [11,12], even to the extent that the individual mRNAs that compose a single gene can be oppositely regulated [13]. We opted to pursue those details on a gene-by-gene basis in future work.

### Enrichment of BC, PC, cancer, and MET annotation among the OI-MET signature genes

We hypothesized that the OI-MET signature gene set represents a model of differential gene expression in MET, common to BC and PC. Therefore, we expected a significant proportion of these genes to have been previously associated with MET, BC, PC, and/or cancer in the literature. To test this hypothesis, we searched both PubMed and PubMed Central (PMC) using an NCBI E-Utilities Perl script to search for each of the 739 genes (using the HGNC gene symbol) AND the phenotype of interest. For example, the query for one of these searches was (“TMEM163”[Text Word] + OR + “tmem163”[Text Word] + OR + “Tmem163”[Text Word]) + AND + (“breast cancer”[Text Word] + OR + “breast neoplasms”[Text Word]). Text word searches for these keywords cast a relatively wide net, capturing essentially any co-occurrence of the gene symbol and the keyword(s) of interest in the manuscript. For instance, a manuscript could mention a given gene and cancer but only in a tangential way, not really associating the gene with cancer. These searches are not very specific because the definition of “text words” is broad, but they provide an upper bound on the number of publications associating each gene with the keyword. We tested the significance of over-representation of each of these annotations in our gene set using a re-sampling approach similar to Li, et al. [14], by randomly selecting 100 sets of 739 genes from the HGNC set, repeating the query for each of these sets, then comparing the number of “hits” in the observed set of 739 genes versus the random sets. We ranked the proportion of genes with hits in the observed set with the proportions of genes in the 100 random sets to produce an empirical p-value.

We are also interested in testing the selected 739 gene set for association with MET, but the text for “mesenchymal to epithelial transition” and “epithelial to mesenchymal transition” are complex, so they are relatively little used in the literature. To overcome this limitation and to balance the high false positive rate expected with the text word searches, we used MeSH [15] (Medical Subject Heading) searches to look for associations between each gene and MET/EMT in the literature. A MeSH term search is more conservative than a text word search, because the MeSH annotation for each manuscript is specific and curated. As such, true gene/keyword associations may be missed, but this provides a lower bound on the number of publications associating each gene with MET. The query for one of these searches was (“TMEM163”[Text Word] + OR + “tmem163”[Text Word] + OR + “Tmem163”[Text Word]) + AND + (“epithelial-mesenchymal transition”[MeSH Terms]). To test the significance of these results we used a contingency table (count of “hits” in the 739 gene see vs the count of “hits” for all genes) to calculate a χ^2^ value and corresponding p-value.

PubMed is a valuable resource for finding text on genes related to cancer in the biomedical literature but not all of PubMed is searchable. PMC is another valuable source of text relating genes to cancer, but it is a less complete collection of manuscripts than PubMed - only ones that are entirely open source. Therefore, we used both the PubMed and PMC databases for our search. In both cases, we compare the proportion of genes associated with each of the keywords in the 739 gene OI-MET signature set, versus the proportion genes associated with each of the keywords for all 36,973 HGNC gene symbols. Notably, many genes have aliases that do not match the HGNC symbol. In that sense, our literature search is conservative because it misses associations between gene and keyword where the gene is not identified by HGNC symbol. Another important consideration is that the literature includes genes that are extensively studied, others that are not as well studied, and some that are essentially unstudied. The genes that are unstudied do not show up in manuscripts, though they may be included in both sets of genes that we studied.

In Table 1, assessing the upper bounds on gene associations with BC, PC, and cancer in the PubMed [text words] search, we see that ~30.9% to 70.5% of genes in the OI-MET signature set are associated with the tested keywords. The equivalent percentages are ~91.9% to 95.1% of genes in the PMC search. For all six tests the empirical p-value is < 0.01. These results are consistent with the OI-MET signature set having a high concentration of BC, PC, and cancer associated genes. It also is consistent with the OI-MET set being a useful model for differential gene expression in BC, PC, and cancer. Assessing the lower bounds on association of the OI-MET gene set with MET/EMT, we find that the MeSH queries in PubMed and PMC show, respectively, ~12.3% and 39.5% of the OI-MET genes as being associated with MET in the literature. Comparing this to the same queries for all genes, we find a significant enrichment for MET associated genes in the OI-MET signature set. For the PubMed comparison, the enrichment is more than 4.5 fold (12.3% vs 2.7%), with a p-value < 0.0001. For the PMC comparison, the enrichment is more than 8.5 fold (38.5% vs 4.5%), also with a p-value < 0.0001. Both of these results are statistically significant, and the fold changes are likely to be biologically relevant, consistent with the OI-MET signature gene set being a useful model for differential gene expression in MET.

**Table 1:** PubMed and PMC searches for OI-MET genes and cancer, BC, PC, and MET

**For 739 OI-MET genes, number found in:**	**PubMed queries for**	**% PubMed**	**p-value**	**PMC queries for**	**% PMC**	**p-value**
**(“cancer”[Text Word] + OR + “neoplasms”[Text Word])**	521	70.5%	< 0.01	703	95.1%	< 0.01
**(“breast cancer”[Text Word] + OR + “breast neoplasms”[Text Word])**	344	46.5%	< 0.01	699	94.6%	< 0.01
**(“prostate cancer”[Text Word] + OR + “prostate neoplasms”[Text Word])**	228	30.9%	< 0.01	679	91.9%	< 0.01
**(“epithelial-mesenchymal transition”[MeSH Terms])**	91	12.3%	< 0.0001	292	39.5%	< 0.0001
**For All 36,973 HGNC Genes, Number found in:**	**PubMed queries for**			**PMC queries for**	**% PMC**	
**(“epithelial-mesenchymal transition”[MeSH Terms])**	995	2.7%		1669	4.5%	

“Cancer”, “breast cancer”, and “prostate cancer” text word searches show that a high proportion of OI-MET genes are associated with these concepts in the literature. “Epithelial-mesenchymal transition” MeSH term searches show a significant enrichment of this annotation in the OI-MET set, relative to all genes: 12.3% ÷ 2.7% = 4.6 Fold Enrichment for PubMed; 39.5% ÷ 4.5% = 8.8 Fold Enrichment for PMC; both with p-value < 0.0001.

### OVOL TF targets in OI-MET

The set of 739 genes in the OI-MET set were all significantly differentially expressed in response to OVOL expression. As such, we tested whether they could all be direct targets of the OVOL TFs. Using the Genomatix Genome Analyzer’s (GGA) Gene2Promoter [16] function, we found 4,102 promoter sequences associated with the mRNAs coded by the 739 genes in the common OI-MET signature. We searched these promoter sequences for OVOL binding motifs using GGA’s MatInspector [17] function, with default parameter settings, and found that only 1,467 of the 4,102 promoters had one or more OVOL binding motifs. This result suggests that, while the OVOLs induced differential expression of all of these genes, the effect must be indirect for at least two thirds of the OI-MET genes.

### Enrichment testing by ConceptGen

Since the OVOLs’ effects on gene expression in MET are not direct, we sought to understand the direct systems involved in OI-MET using ConceptGen [18] enrichment testing. This search is complementary to the literature search, based on annotation derived from the literature. Of the 739 genes in the OI-MET signature, 727 uniquely mapped to Entrez GeneIDs using the DAVID [19] ID converter. Of these 727 genes, 719 had annotation in at least one category in ConceptGen. In the most significant block of annotation (Additional file 2), we found enrichment for annotation consistent with MET (e.g. “Epithelial Cells” FDR 1.65E-13, “Response to Wounding” FDR 6.10E-13), and with cancer metastasis (e.g. “Cell Movement” FDR 1.61E-08, “Cell Adhesion” FDR 6.48E-08). As we found in the literature search, these results are consistent with the OI-MET signature being a useful model for characterizing differential gene expression in MET associated with BC and PC progression.

Also consistent with the observation that the OVOL TFs likely regulate gene expression in OI-MET, ConceptGen found enrichment for “Signal Transduction” FDR 1.75E-10 and “Gene Expression Regulation, Neoplastic” FDR 2.06E-08. This led us to pursue the details of gene expression regulation in this annotation, and we found enrichment for regulation of gene expression by five TFs: AP-1 (AP1) FDR 1.16E-04, c-Jun (JUN) FDR 5.47E-03, NF-kappa B (NFKB1) FDR 4.78E-05, STAT1 FDR 3.40E-02, and STAT3 FDR 1.07E-02 (Additional file 2). The genes in the AP1 and JUN sets are annotated as TransFac [20] (direct binding) targets while the genes in the NFKB1, STAT1, and STAT3 sets are annotated as being associated with the TFs in MeSH [15] annotation. Notably, though ConceptGen identified one set of genes as being TransFac targets for AP1 and an overlapping set for JUN, AP-1 is a dimer [21,22] of subunits from the FOS and JUN gene families (c-Fos, FosB, Fra-1, Fra-2, c-Jun, JunB, JunD), so we collapsed the AP-1 and c-Jun sets into a single set of genes targeted by the AP1 dimer. Note that NF-kappa B is also a dimer composed of subunit pairs [23] (NF-kB1, NF-kB2, c-Rel, RelA, and RelB). TFs routinely work together as homo- or hetero-dimers, or in modules composed of multiple TF complexes [24]. This observation, along with our previous observation that the OVOLs influence the OI-MET gene set indirectly, led us to hypothesize that the OVOL TFs impact MET through interaction with, or in collaboration with, these other four TFs.

### OVOLs use complex mechanisms to regulate AP1, STAT1, STAT3, and NFKB

We used the expression data derived from this experiment to test this hypothesis (Table 2). We assessed the effects of the OVOLs on expression of AP1, STAT1, STAT3, and NFKB. For some genes, “Expression Level” is assayed at only the gene level. Other genes are derived from multiple mRNAs, so they are assayed at the isoform level then the data are aggregated to produce gene level expression information. We looked for up-regulation (at least one observation of Fold Change ≥ 1.5) or down-regulation (at least one observation of FC ≤ 0.67). We looked for isoform switching, meaning that one isoform is up-regulated and another isoform is down-regulated (switch), as a potential regulatory mechanism in genes where more than one mRNA was tested. Within the AP1 (FOS/JUN) group, the FOS genes are regulated in both BC and PC, at both the gene and isoform levels (e.g. FOSB). The individual members of the JUN family are regulated at the gene level, similar to the way isoform switching is used in the FOS group. Within the STAT group (Stat1/Stat3), isoform level regulation is employed in BC while both isoform switching and gene level regulation are employed in PC. Within the NFKB group, gene level regulation is employed.

**Table 2:** **Regulation of AP1**, **STAT1**, **STAT3**, **and NFKB expression by OVOLS**

			**BC fold change**				**PC fold change**			
	**Gene**	**Expression level**	**OVOL1**	**OVOL2**	**OVOL1&2**	**Up/Down switch**	**OVOL1**	**OVOL2**	**OVOL1&2**	**Up/Down switch**
**AP1**	**FOS (c-Fos)**	**Gene**	1.4	1.4	1.4		1.0	0.8	1.0	
	**FOSB**	**Gene**	1.0	2.1	1.1	**Up**	0.7	0.5	0.4	**Down**
		**NM**_**006732**	2.6	0.0	0.0	**Switch**	10.0	10.0	10.0	**Switch**
		**NM**_**001114171**	0.9	2.3	1.1		0.7	0.5	0.4	
	**FOSL1 (Fra-1)**	**Gene**	0.9	0.9	0.8		1.1	1.0	0.7	
	**FOSL2 (Fra-2)**	**Gene**	0.7	0.7	0.6	**Down**	0.8	1.2	1.1	
	**JUN (c-Jun)**	**Gene**	1.0	0.9	0.8		0.5	0.5	0.5	**Down**
	**JUNB**	**Gene**	1.5	2.2	2.1	**Up**	1.2	1.9	1.4	**Up**
	**JUND**	**Gene**	1.1	1.1	1.3		1.2	1.6	1.2	**Up**
**STAT**	**STAT1**	**Gene**	1.1	1.3	1.0		1.0	1.4	2.0	**Up**
		**NM**_**007315**	1.1	1.3	1.0		1.1	1.4	1.9	**Up**
		**NM**_**139266**	1.2	1.3	1.1		0.8	1.7	2.2	**Up**
	**STAT3**	**Gene**	0.9	1.2	0.9		0.0	0.4	6.3	**Up/down**
		**NM**_**139276**	0.5	1.6	0.9	**Up/down**	0.0	0.4	0.0	**Switch**
		**NM**_**003150**	0.8	1.2	0.9		0.0	1.0	10.0	
		**NM**_**213662**	1.0	1.2	0.9		0.1	1.2	8.6	**Up/down**
**NFKB**	**NFKB1 (NFkB1)**	**Gene**	1.0	1.3	1.2		1.0	1.1	1.0	
		**NM**_**003998**	1.0	1.3	1.2		1.3	1.1	1.2	
		**NM**_**001165412**	1.0	1.2	1.2		0.8	1.0	0.9	
	**NFKB2 (NFkB2)**	**Gene**	0.9	1.5	1.2	**Up**	1.4	1.7	0.8	**Up/down**
		**NM**_**001077494**	0.9	1.5	1.0	**Up**	1.9	3.8	0.8	**Up**
		**NM**_**002502**	0.7	1.6	0.9	**Up**	1.3	1.9	0.7	**Up**
		**NM**_**001261403**	0.9	1.4	0.9		1.4	1.4	0.8	
	**REL (c-Rel)**	**Gene**	1.2	1.3	1.1		1.6	2.2	1.6	**Up**
	**RELA**	**Gene**	1.0	1.1	1.1		0.9	0.9	0.8	
		**NM**_**021975**	1.0	1.0	1.0		1.0	1.0	1.0	
		**NM**_**001145138**	1.0	1.1	1.1		0.9	0.9	0.8	
	**RELB**	**Gene**	0.8	1.5	1.1	**Up**	1.3	2.4	1.0	**Up**

We assessed the effects of the OVOLs on expression of AP1, STAT1, STAT3, and NFKB based on our RNA-Seq data for both BC and PC. We looked for up-regulation (at least one observation of Fold Change ≥ 1.5), down-regulation (at least one observation of FC ≤ 0.67), or isoform switching (switch). Within the AP1 (FOS/JUN) group, the FOS genes are regulated in both BC and PC, at both the gene and isoform levels (e.g. FOSB). The individual members of the JUN family are regulated at the gene level, similar to the way isoform switching is used in the FOS group. Within the STAT group (Stat1/Stat3), isoform level regulation is employed in BC while both isoform switching and gene level regulation are employed in PC. Within the NFKB group, gene level regulation is employed.

### Development of the OI-MET-TF network

To help us understand the potential roles of these TFs in OI-MET, we used GeneGo MetaCore [25] to model the networks of interactions among each of the gene sets annotated as targets for the four TFs (AP1, NFKB1, STAT1, STAT3), and the OVOL TFs. In each of these subsets, we included the genes annotated as TF targets in the ConceptGen analysis, as well as the TF, and used parameter settings to produce the most parsimonious models possible (i.e. the simplest models that include all of the input genes).

**Figure 2:**
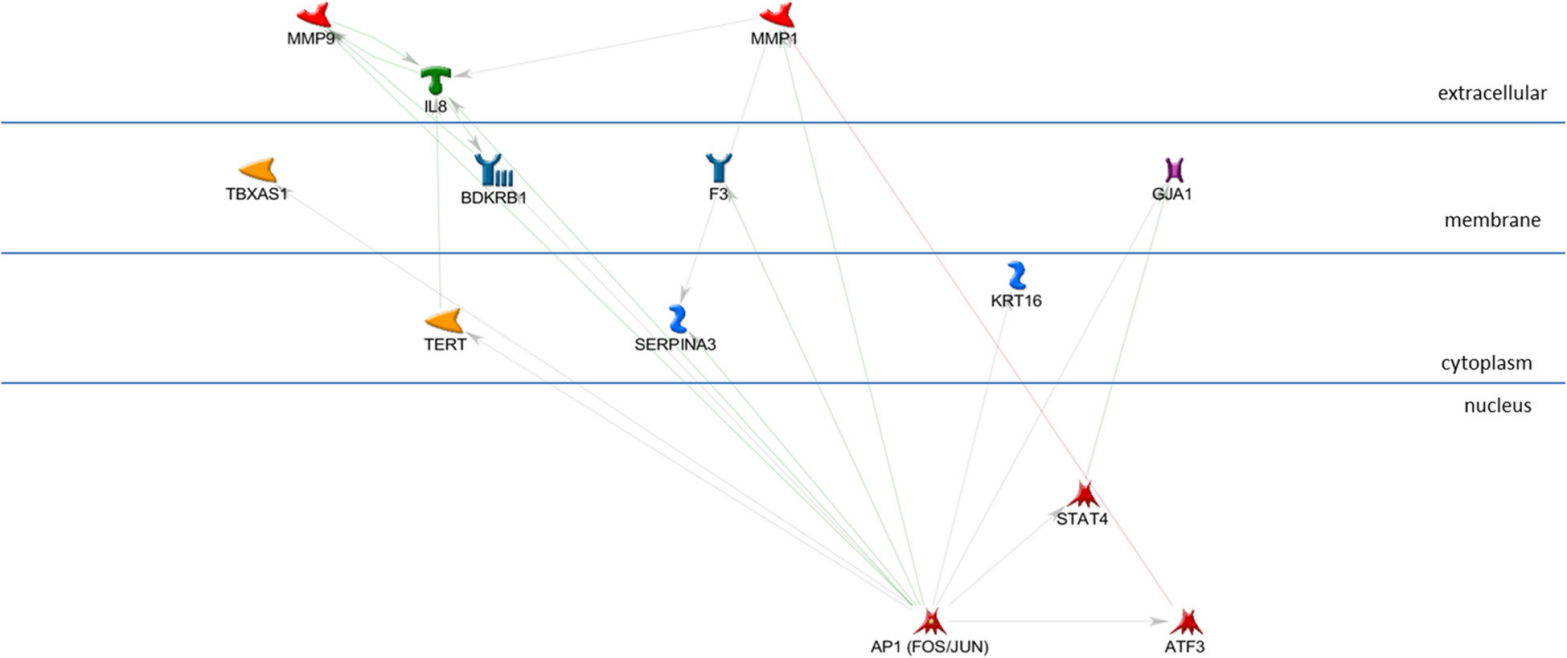
**AP1 sub-network.** The AP1 network, including FOS, JUN and their TransFac annotated targets. All of the nodes in this network are direct targets of AP1.

**Figure 3:**
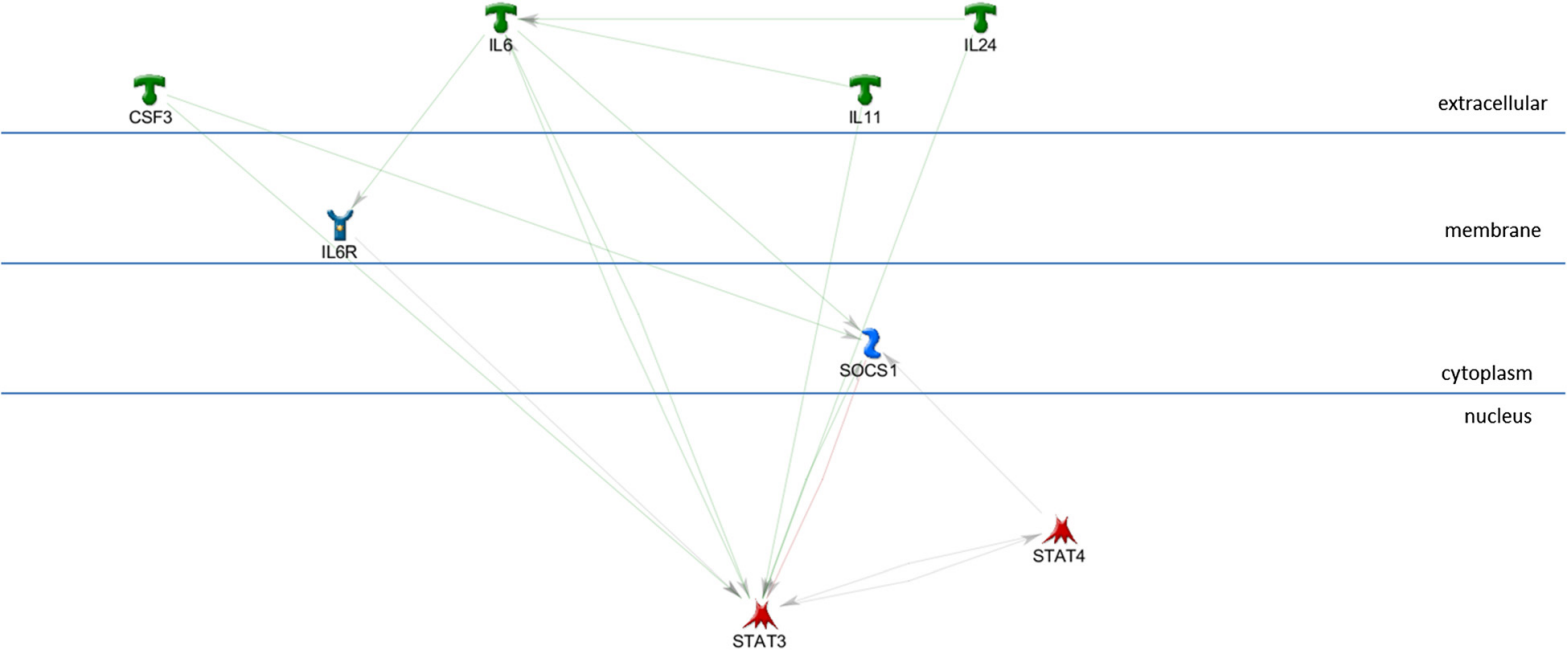
**STAT3 sub-network.** The STAT3 network, including STAT3 and its MeSH annotated targets. All of the nodes are connected, though MeSH association does not necessarily indicate direct binding.

**Figure 4:**
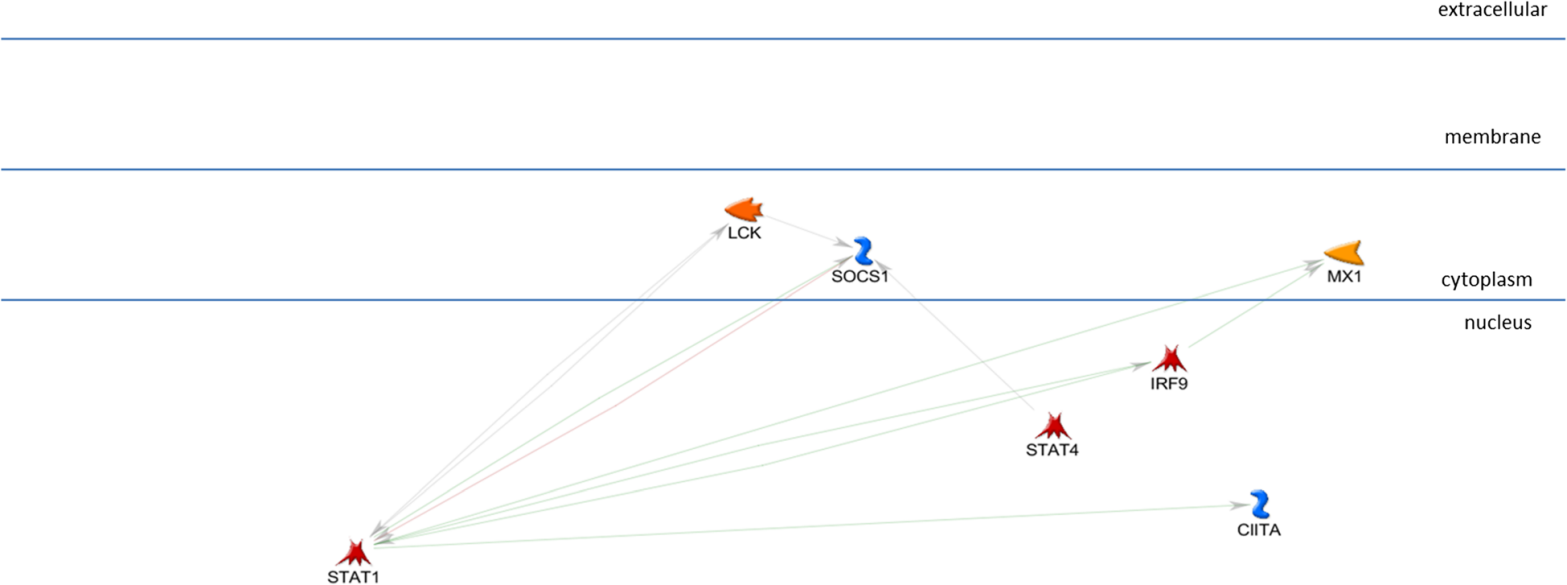
**STAT1 sub-network.** The STAT1 network, including STAT1 and its MeSH annotated targets. All of the nodes are connected, though MeSH association does not necessarily indicate direct binding.

**Figure 5:**
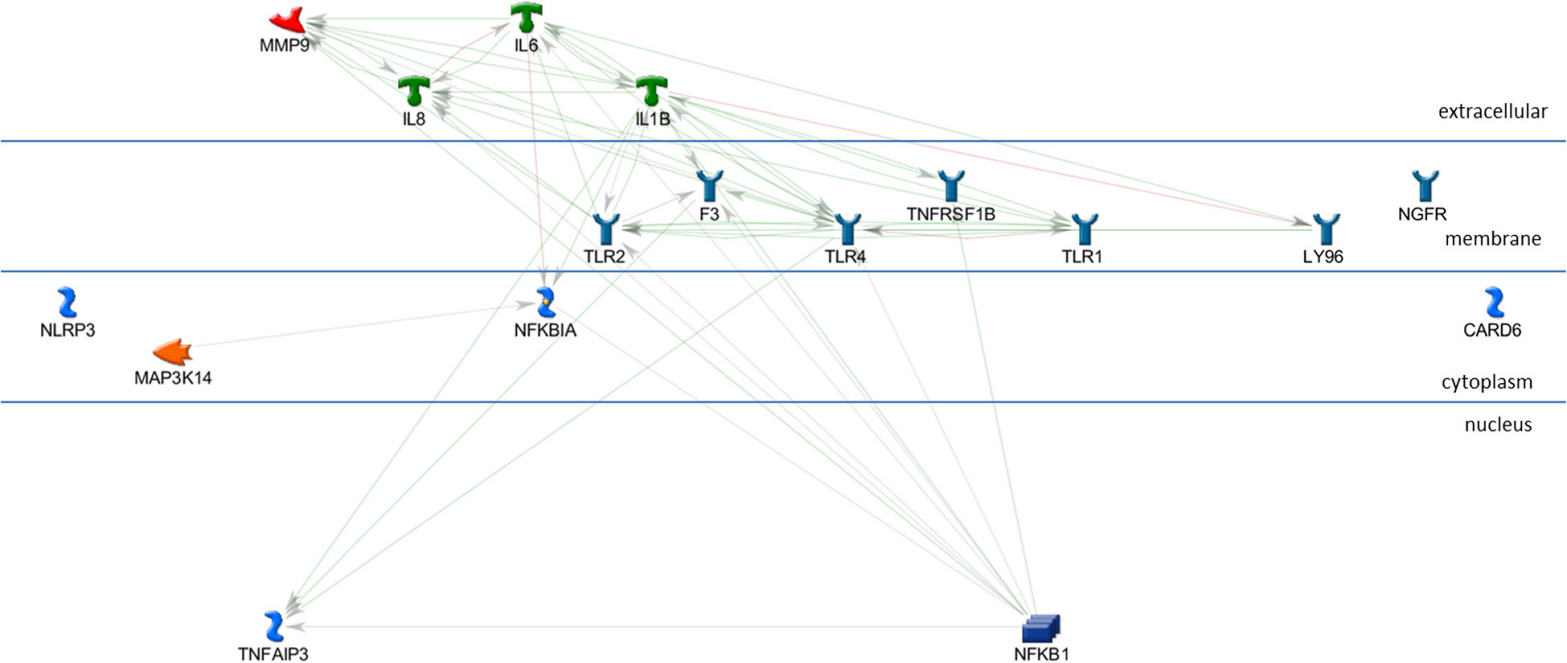
**NFKB1 sub-network.** The NFKB1 network, including NFKB1 (multiple modules) and its MeSH annotated targets. Not all of the nodes are connected in this network.

**Figure 6:**
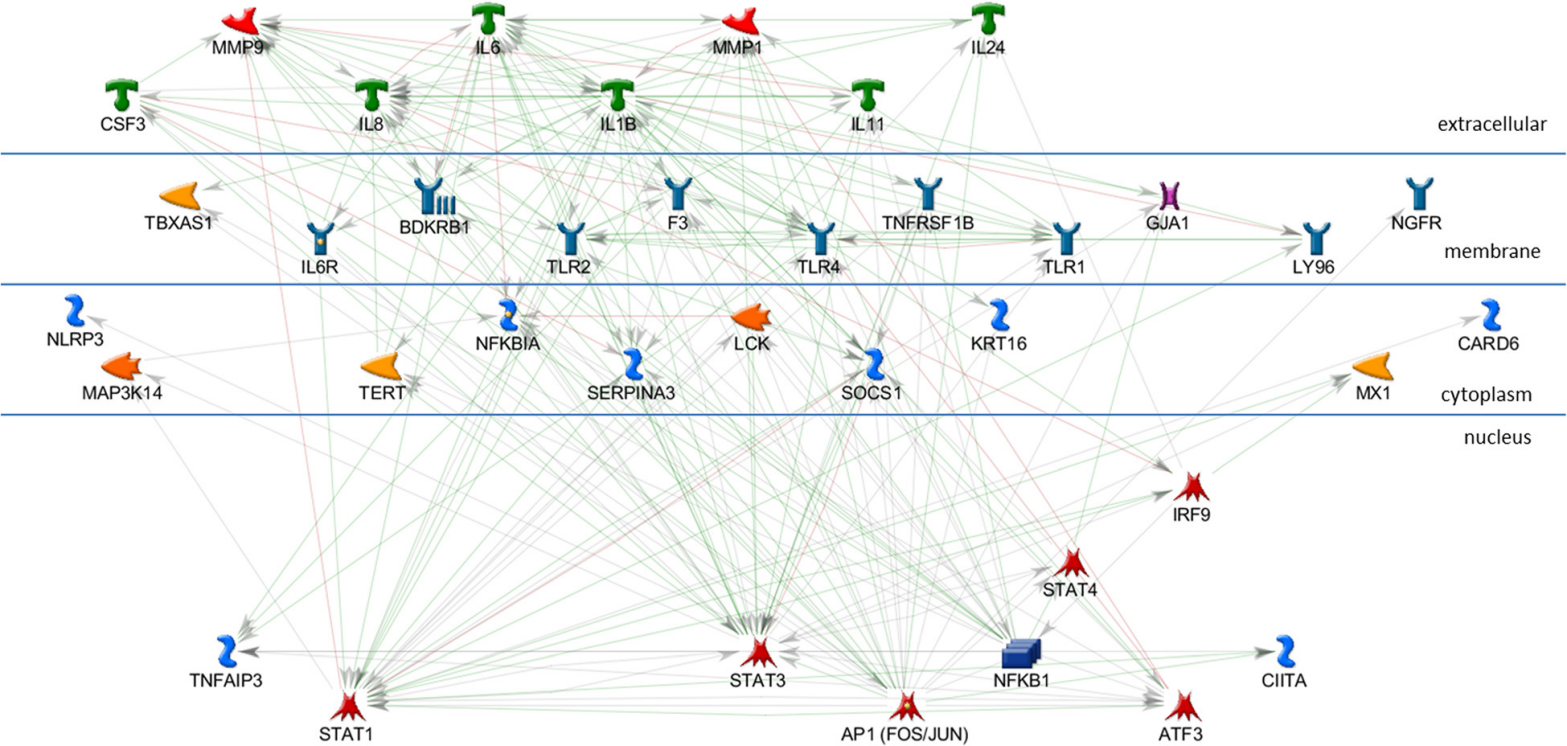
**Combined AP1, STAT3, STAT1, and NFKB1 sub-network.** In the combined network, all of the nodes are connected.

The AP1, STAT3, and STAT1 networks each include all of the input genes in a very simple, parsimonious, network (Figures 2, 3, and 4). This is consistent with what was expected for the AP1 network because the genes in this set are annotated as being direct AP1 binding targets in TransFac annotation. Note that Additional file 3 is the key for interpreting GeneGo graphics and that the icon labeled “AP1 (FOS/JUN)” represents the dimer of FOS and JUN gene family members in a single icon. Genes in the STAT1 and STAT3 networks are found in MeSH annotation and, while all the genes are in the network, they are not all direct targets of the TF. The NFKB1 network (Figure 5), also derived from MeSH annotation, illustrates that the annotation does not necessarily indicate direct interaction with the TF. Rather, using the same parameter settings as for the other networks, NGFR, CARD6, and NALP3 are disconnected genes. Also, this network includes NFKBIA, which interacts closely with, but is distinct from the NFKB1 dimer. Note that GeneGo used two icons for NFKB1, but we collapsed them into a single rectangular icon in this graphic. It’s possible that a more complex (less parsimonious) NFKB1 network would connect all the genes in the NFKB1 set, but our hypothesis is that these four TFs work together in regulating the genes differentially expressed in OI-MET. Therefore, we developed the network for the combined set of genes targeted by the four TFs using the parameter settings for the parsimonious network. The network we found (Figure 6) is consistent with this hypothesis; it connects all the genes and includes only one gene that was not part of the input set (the aforementioned NFKBIA).

**Figure 7:**
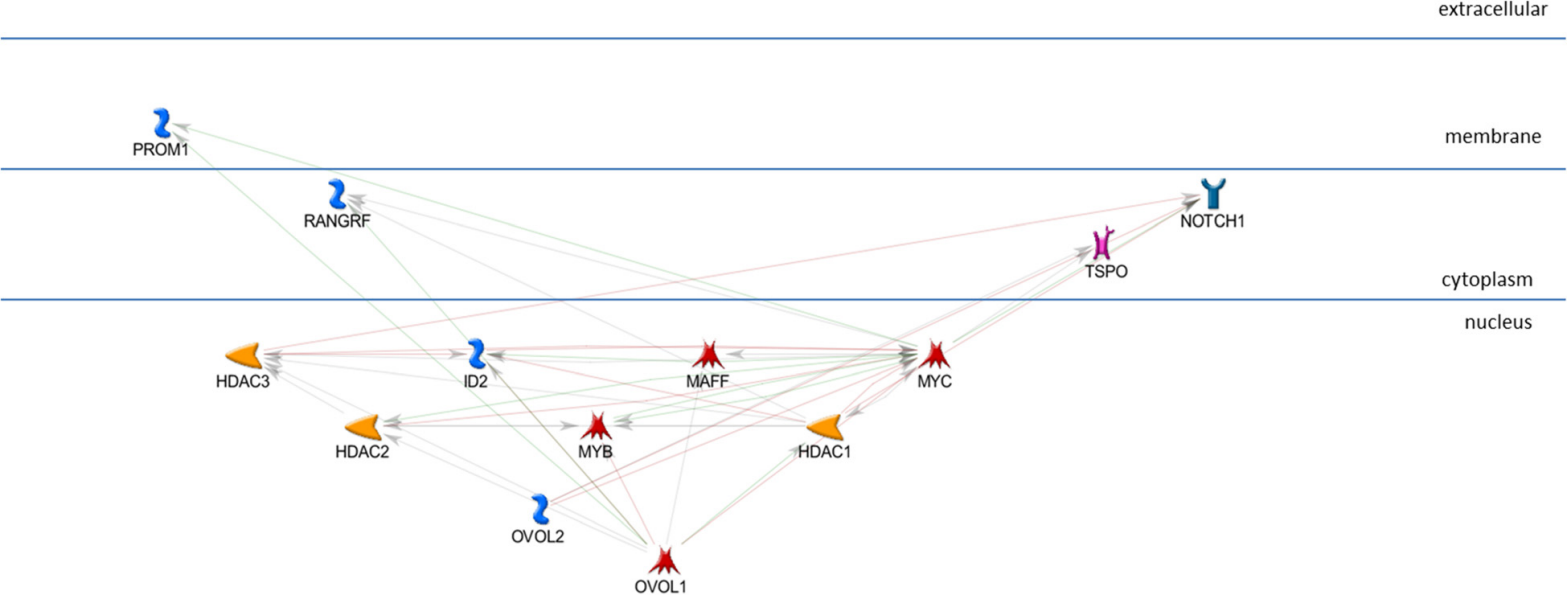
**OVOL1, OVOL2, and their direct targets sub-network.** We selected the direct targets of the OVOLs to make the sub-network consistent with the sub-networks of the other TFs.

**Figure 8:**
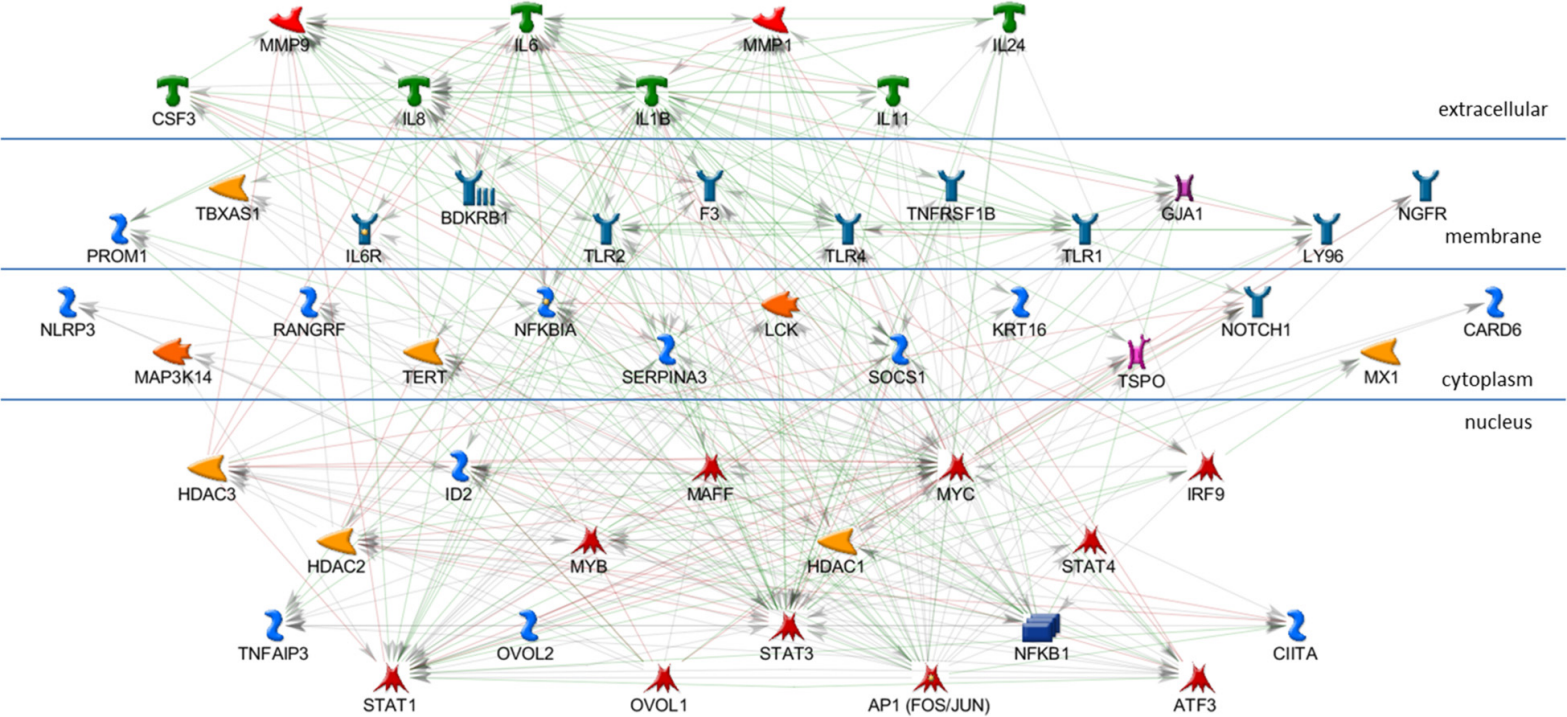
**OI-MET-TF network.** The combined AP1, STAT3, STAT1, NFKB1, and OVOLs network. The network is parsimonious in that it connects all the nodes and includes only one gene not part of the input set.

While this network is highly consistent with the cooperative regulation of these genes by this set of four TFs in OI-MET, it does not yet explain the effects of the OVOL TFs. To understand how the OVOLS impact this network, we created a network similar to those of the four TFs enriched in the ConceptGen analysis. Consistent with the other networks, we focused on the targets of OVOL1 and OVOL2 (Figure 7). In the OVOL sub-network, OVOL1 shows eight annotated targets while OVOL2 shows only three annotated targets, with MYC as the single target common to the two OVOLs. As we did in developing the network in the previous step, we added the OVOL targets sub-network to the AP1, NFKB1, STAT1, and STAT3 network and found that the OVOLs have multiple indirect influences on this combined network (Figure 8). We call this the OI-MET-TF network because it focuses on only the genes annotated as being targets of the four TFs enriched in ConceptGen data, plus the OVOLs and their targets.

We hypothesized that the OVOLs work indirectly in influencing the expression of the OI-MET genes. Based on this hypothesis, we would expect the OI-MET-TF gene set to form a connected and parsimonious network. Consistent with this hypothesis, every gene in the OI-MET-TF model is included in the network and there are no disconnected nodes. The network is parsimonious, as only a single gene (NFKBIA) that is not part of the input gene set is included in this network. The GeneGo interactions annotation shows that the five TFs of interest do work together to regulate the combined set of genes. For example, NGFR, CARD6, and NALP3 are disconnected nodes in the NFKB1 network but we now see that NGFR and NALP3 are targets of AP1, while CARD6 is a target of STAT1. The OI-MET-TF network shows that many genes annotated as being targets of one of the five TFs of interest are also targets of one or more of the other TFs. For example, SOCS1 is a target of STAT3, STAT1, and AP1. Based on GeneGo interactions data, we find that one gene is regulated by 4 of the TFs of interest, four genes are regulated by 3 of them, and twenty genes are regulated by 2 of them.

We further hypothesized that the OI-MET-TF network model is useful in understanding gene expression changes in MET common to BC and PC. Therefore, we would expect a significant proportion of the genes in the network to be associated with BC, PC, cancer, and MET. As we did with the 739 gene OI-MET gene set, we searched PubMed and PMC using an NCBI E-Utilities Perl script to search for each gene (using the HGNC gene symbol) and phenotype of interest[text words], as well as “epithelial-mesenchymal transition”[MeSH].

As shown in Table 3, for all six tests the empirical p-value is < 0.01, and at least 48 of the 52 (92%) genes in the OI-MET-TF network model are already associated with each of these key MET and cancer related concepts in PMC, consistent with the network being a useful model for analysis of gene expression in MET and cancer. The evidence is less strong in PubMed but, even in that case, more than 69% the genes are MET and cancer related. While we found the 739-gene OI-MET signature set to be significantly associated with each of these cancer and MET related terms, we find the enrichment is even greater in the OI-MET-TF model. Again, assessing the lower bounds on association of the OI-MET gene set with MET/EMT, we find that the MeSH queries in PubMed and PMC show, respectively, ~40.4% and 73.1% of the OI-MET-TF model genes as being associated with MET in the literature. Also, comparing this to the equivalent queries for all genes, we find a significant enrichment for MET associated genes in the OI-MET-TF signature set. For the PubMed comparison, the enrichment is more than 15 fold (40.4% vs 2.7%) with a p-value < 0.0001. For the PMC comparison, the enrichment is more than 16 fold (73.15 vs 4.5%), also with a p-value < 0.0001. Both of these results are consistent with the OI-MET-TF model being useful for understanding the regulation of differential gene expression in MET.

**Table 3:** **PubMed and PMC searches for OI**-**MET**-**TF genes and cancer**, **BC**, **PC**, **and MET**

**For 52 OI-MET**-**TF genes, number found in:**	**PubMed queries for**	**% PubMed**	**p-value**	**PMC queries for**	**% PMC**	**p-value**
**(“cancer”[Text Word] + OR + “neoplasms”[Text Word])**	45	86.5%	< 0.01	48	92.3%	< 0.01
**(“breast cancer”[Text Word] + OR + “breast neoplasms”[Text Word])**	47	90.4%	< 0.01	49	94.2%	< 0.01
**(“prostate cancer”[Text Word] + OR + “prostate neoplasms”[Text Word])**	36	69.2%	< 0.01	48	92.3%	< 0.01
**(“epithelial-mesenchymal transition”[MeSH Terms])**	21	40.4%	< 0.0001	38	73.1%	< 0.0001
**For All 36,973 HGNC Genes, Number found in:**	**PubMed queries for**			**PMC queries for**	**% PMC**	
**(“epithelial-mesenchymal transition”[MeSH Terms])**	995	2.7%		1669	4.5%	

As with the OI-MET gene set “cancer”, “breast cancer”, and “prostate cancer” text word searches show that a high proportion of OI-MET-TF genes are associated with these concepts in the literature. For all six tests the empirical p-value is < 0.01. “Epithelial-mesenchymal transition” MeSH term searches show an even more significant enrichment of this annotation in the OI-MET set, relative to all genes: 40.4% ÷ 2.7% = 15.2 Fold Enrichment for PubMed; 73.1% ÷ 4.5% = 16.4 Fold Enrichment for PMC; both with p-value < 0.0001.

As we tested the OI-MET signature gene set with both literature searches and ConceptGen, we tested the OI-MET-TF model with both literature searches, above, and GeneGo’s built in enrichment algorithm for disease processes (Table 4). Note that, while ConceptGen provides FDR values to account for multiple testing, the GeneGo table presents uncorrected p-values. In the OI-MET-TF model, we find over-representation of rheumatologic diseases pathobiology - immune/inflammation, joint, dry eye, and dry mouth annotated genes. “Inflammation” and “Wounds and Injuries” are consistent with ConceptGen enrichment in the common OI-MET set (Additional file 2).

**Table 4:** **MetaCore enrichment for disease processes in the OI**-**MET**-**TF gene set**

**Disease phenotype**	**% of genes annotated for:**	**p-Value**
**Pathologic processes**	84.0	8.6E-46
**Rheumatic diseases**	72.0	9.2E-44
**Connective tissue diseases**	72.0	4.9E-40
**Arthritis**	70.7	3.2E-41
**Joint diseases**	70.7	7.1E-41
**Arthritis, rheumatoid**	69.3	1.8E-42
**Inflammation**	62.7	1.8E-46
**Dry eye syndromes**	54.7	3.4E-43
**Lacrimal apparatus diseases**	54.7	3.4E-43
**Sjogren’s syndrome**	53.3	1.4E-43
**Xerostomia**	53.3	1.4E-43
**Salivary gland diseases**	53.3	5.4E-40

Disease phenotypes consistent with cancers, as well as inflammation are enriched in annotation for the OI-MET-TF set. (Note that p-values are not adjusted for multiple hypothesis tests).

### Prioritizing drug targets

**Figure 9:**
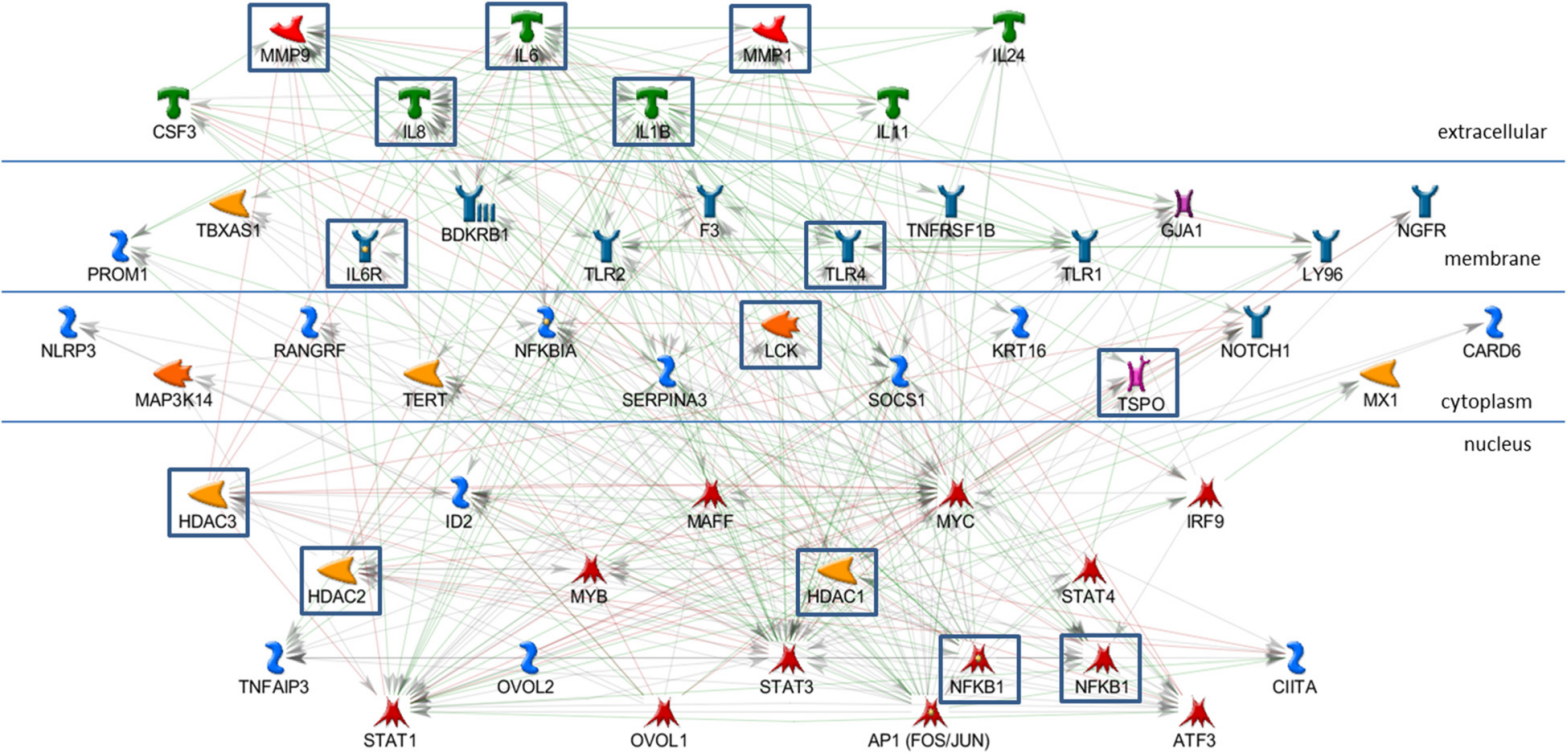
**Drug targets in the OI-MET-TF network.** Drug targets are boxed. Fourteen genes in the network are documented drug targets.

Bioinformatics analyses like the one offered here have the power to provide evidence capable of intelligently guiding selection of the most promising drug combinations to test from an otherwise near-infinite possible number of synergies between approved and in-approval drugs [26]. Using GeneGo’s MetaDrug database, we prioritized drug/gene target combinations in this network for follow-on testing, emphasizing the potential clinical/translational relevance of this work (Figure 9). Note that we expanded NFKB1 in this graphic to reveal the two groups, both of which are drug targets. There are 80 drug/gene target combinations (Additional file 4) based on annotation in the GeneGo MetaDrug [25] database (14 genes crossed with 34 drugs). Status of these drugs includes a combination of Phase I, II, and III clinical trials, as well as drugs approved for use in humans. The current applications include drugs used in cancer treatment, across a spectrum of cancer types, as well as a range of other diseases (e.g. bronchitis, pulmonary disease, arthritis, psoriasis). For drugs with known cancer and other applications, network interactions could help identify, prevent, or explain side effects. Novel cancer therapies could also be derived for known drugs that are used for other diseases.

Among recently approved novel cancer chemotherapeutics are those which inhibit HDAC activity [27], and those that inactivate of NFKB signaling with proteasome inhibitors [28]. A growing number of early clinical trials are exploring attempting to synergize the effects of HDAC inhibitors and NFKB interfering proteasome inhibitors to treat solid tumors, with variable reported success [29,30]. The prominence in this network model of HDACs as direct partners for OVOL function, and the NFKB signaling pathway as a regulator of MET-associated genes, offer suggestions that this type of synergistic approach, combining HDAC inhibitors (such as vorinostat) with proteasome inhibitors (such as bortezomib) might have value in advanced prostate and breast cancer. Intriguingly, vorinostat and bortezomib were recently shown to synergistically inhibit the growth of prostate cancer cell lines and suppress tumor growth in murine xenograft models [31]. As future therapeutic agents are developed, this model will continue to provide guidance, potentially allowing identification of those future agents with mechanisms of action that might be particularly efficacious against OVOL-related contributions to metastatsis. In addition, while essentially all cancer therapeutics have significant off target effects, this model may be used to predict off target effects for both current and future therapies, allowing clinicians to better understand and minimize these complications in cancer therapy.

### Indirect action of the OVOL TFs

**Figure 10:**
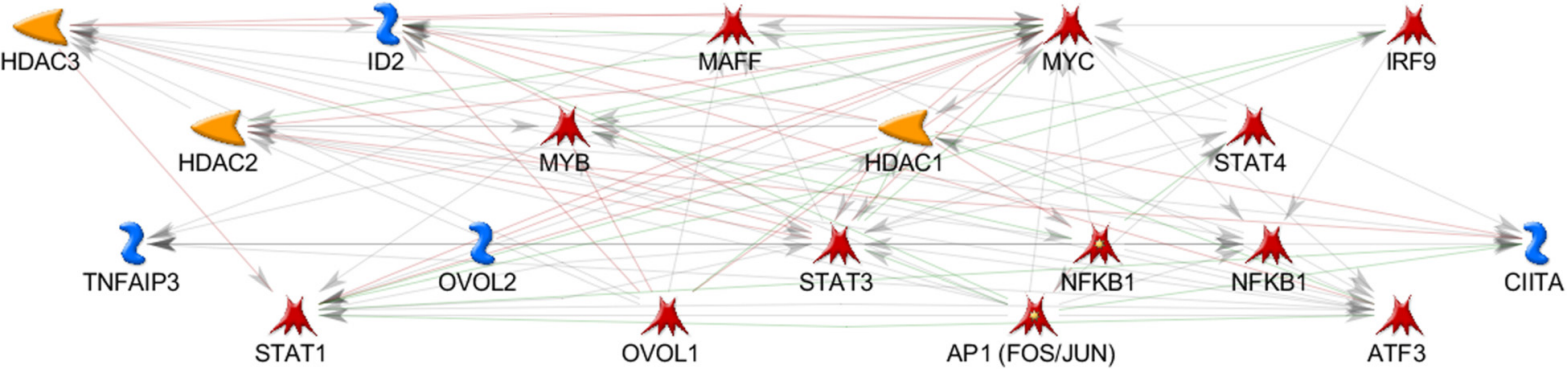
**Nucleus only, OI-MET-TF network.** Focusing only on the nucleus, it is evident that the OVOL TFs indirectly influence expression in the network, setting off a cascade of gene expression.

As seen in the OI-MET-TF model, the effects of the OVOL TFs are complex and they interact directly with only a small number of genes in the network. Focusing on only the nuclear proteins from that network (Figure 10), both OVOLs regulate MYC while OVOL1 also directly regulates MAFF and MYB (transcription factors), ID2 (an inhibitor of DNA binding), plus HDAC1, HDAC2, and HDAC3 (histone deacetylases). These interactions are consistent with the hypothesis that OVOLs influence OI-MET gene expression indirectly, setting off a cascade of downstream effects. In this model, the signal propagates from the OVOLs, through MYC, MYB, and MAFF. This signal would be modulated by ID2 and the HDACs, which subsequently regulate TNFAIP3, AP1, STAT1, STAT3, STAT4, NFKB1, IRF9, ATF3, and CIITA. Note that the OI-MET-TF model suggests that MAFF, ATF3, MYC, MYB, STAT4 and IRF9 are potentially important TFs in this regulatory cascade, in addition to the TFs identified by ConceptGen (AP1, NFKB1, STAT1, and STAT3) and the OVOLs. Using publicly available ChIP-Seq data, we test the validity of this hypothesized cascade, below.

### Reflection back to the OI-MET gene expression signature

Keeping in mind that the OI-MET-TF network is necessarily simplistic, this network is strongly consistent with the hypothesis that the OVOLs regulate MET in concert with the other four TFs. However, since the roles of the other four TFs were suggested by enrichment of annotation in the OI-MET signature gene set, we hypothesized that the effects of these TFs from the OI-MET-TF model are consistent across the larger OI-MET signature set. We also observed that, in addition to the four TFs identified by ConceptGen, MAFF, ATF3, MYC, MYB, and IRF9 could be important in this regulatory cascade. Using GGA’s MatInspector function, we searched the 4,102 promoters from the OI-MET gene set, looking for individual binding sites for these promoters (Table 5). Based on the number of sequences with one or more binding sites for each of these TFs, and comparing to the frequency expected for all promoters, we find that NFKB, MYC, and to a lesser extent, MAFF motifs are over-represented in these promoters. Note that, while these motifs are over-represented, the modest values of over-representation make their biological relevance subject to interpretation. Equally, as noted below, the presence of single motifs is not a strong indicator of regulatory control. The proportion of promoters with the other motifs is not significantly different from the proportion expected for a random set of promoters at a significance threshold of p-value ≤ 0.05.

**Table 5:** **Promoters for the OI**-**MET gene set**, **proportion with TF motifs tested singly and compared to all promoters**

	**AP1**	**MAFF**	**ATF3**	**MYC**	**IRF6**	**NFKB1**	**OVOL**	**STAT**	**MYB**
**Motif name**	**V$AP1F**	**V$AP1R**	**V$CREB**	**V$EBOX**	**V$IRFF**	**V$NFKB**	**V$OVOL**	**V$STAT**	**V$MYBL**
**# sequences with motif**	1380	3193	3267	2472	2696	2386	1467	3191	2873
**p-value**	5.9E-01	**3.1E-02**	7.7E-01	**3.8E-08**	1.0E + 00	**3.9E-27**	1.0E + 00	9.9E-01	1.0E + 00
**Observed proportion**	33.6%	77.8%	79.6%	60.3%	65.7%	58.2%	35.8%	77.8%	70.0%
**Expected proportion**	33.8%	76.6%	80.1%	56.1%	70.5%	49.8%	41.1%	79.4%	74.7%
**Fold enrichment**	0.995	**1.016**	0.994	**1.074**	0.932	**1.168**	0.870	0.980	0.938

When tested singly, only MAFF, MYC, and NFKB motifs are enriched in promoters from the OI-MET gene set.

Values in bold are significant at the p-value < 0.05 level.

Since TFs generally work in pairs or modules, we searched the 4,102 promoter sequences for all pairs of motifs derived from these individual motifs using GGA’s RegionMiner module (Table 6). RegionMiner compared the proportion of promoters with each motif pair in the 4,102 promoters versus the proportion of promoters with the motif pair in all GGA promoters. This is the observed enrichment in Table 6. For comparison, we calculated the expected representation in this group of 4,102 promoters as the product of fold enrichment for the first motif x the fold enrichment for the second motif. This is the value expected if the motifs were randomly distributed across the 4,102 promoters (Expected enrichment, Table 6). For almost all of these TF pairs, we found approximately the expected number of promoters with the motif pair. However, the V$AP1F/V$EBOX motif pair, corresponding to the AP1/MYC TF pair, showed 1.38 fold enrichment relative to all promoters in the RegionMiner search. Based on our calculation, we would have expected only 1.07 fold enrichment. This difference (observed 433 promoters with the pair vs. expected 314) is the largest in our dataset and is significant at the χ^2^ p-value < 0.01 level. Finding a much greater proportion of promoters with the motif pair than expected by chance suggests that cooperative regulation by AP1 and MYC could be important in the downstream cascade of gene expression regulating MET.

**Table 6:** **Enrichment for binding motifs for TF pairs in the OI**-**MET gene set**

**Motif pair**	**Observed enrichment in OI-MET promoters for motif pair**	**Expected enrichment**	**Difference**
**V$AP1F V$EBOX**	1.38	1.07	0.31
**V$IRFF V$OVOL**	0.91	0.81	0.10
**V$AP1R V$EBOX**	1.18	1.09	0.09
**V$AP1F V$STAT**	1.07	1.00	0.07
**V$AP1R V$STAT**	1.07	1.00	0.07
**V$AP1F V$IRFF**	0.99	0.93	0.06
**V$NFKB V$STAT**	1.20	1.14	0.06
**V$AP1F V$AP1R**	1.04	0.99	0.05
**V$AP1F V$CREB**	1.03	0.99	0.04
**V$IRFF V$STAT**	0.95	0.91	0.04
**V$AP1F V$NFKB**	1.19	1.16	0.03
**V$AP1F V$MYBL**	0.96	0.93	0.03
**V$MYBL V$NFKB**	1.12	1.10	0.02
**V$AP1R V$IRFF**	0.97	0.95	0.02
**V$AP1R V$MYBL**	0.97	0.95	0.02
**V$IRFF V$MYBL**	0.89	0.87	0.02
**V$AP1R V$CREB**	1.02	1.01	0.01
**V$MYBL V$OVOL**	0.82	0.82	0.00
**V$AP1R V$NFKB**	1.18	1.19	−0.01
**V$AP1R V$OVOL**	0.92	0.95	−0.03
**V$MYBL V$STAT**	0.89	0.92	−0.03
**V$OVOL V$STAT**	0.82	0.85	−0.03
**V$EBOX V$STAT**	1.05	1.09	−0.04
**V$AP1F V$OVOL**	0.82	0.87	−0.05
**V$CREB V$NFKB**	1.11	1.16	−0.05
**V$CREB V$IRFF**	0.87	0.93	−0.06
**V$CREB V$STAT**	0.91	0.97	−0.06
**V$IRFF V$NFKB**	1.01	1.09	−0.08
**V$EBOX V$IRFF**	0.92	1.00	−0.08
**V$CREB V$MYBL**	0.85	0.93	−0.08
**V$EBOX V$NFKB**	1.17	1.25	−0.08
**V$CREB V$OVOL**	0.77	0.87	−0.10
**V$NFKB V$OVOL**	0.92	1.02	−0.10
**V$EBOX V$OVOL**	0.81	0.93	−0.12
**V$EBOX V$MYBL**	0.86	1.01	−0.15
**V$CREB V$EBOX**	0.91	1.07	−0.16

The AP1/MYC binding motif pair is much more common in the OI-MET promoters than would be expected if they were independent.

### Testing the OI-MET signature with ChIP-Seq data

Testing for the presence of TF binding motifs is useful in identifying potential regulatory effects in a set of promoters. Enrichment for motifs and, more significantly, enrichment for modules composed of motif pairs, as we did, is potentially even more useful. However, the presence of a binding motif or motif pair does not necessarily mean that the TFs bind, or that they bind under relevant conditions. ChIP-Seq is a high-throughput process for identifying DNA sequences bound by proteins, including transcription factors [32]. To test whether the 4,102 promoters in the OI-MET set bind the TFs of interest in relevant tissues, we downloaded ChIP-Seq data from ChipBase [33]. ChIPBase is a database of transcription factor binding maps, based on publicly available ChIP-Seq data, for cell lines derived from various tissues. Relevant to our study, we were able to download binding data for AP1 (both FOS and JUN), ATF3, MYC, NFKB1, and STAT1 TFs. For each TF, we had zero or more files from cell lines derived from non-cancer tissue (WBC samples from the Coriel repository or HUVEC cells), solid tumors (HeLa or HepG2 cell lines), and leukemia (K562 cell line).

Throughout the earlier steps in the analysis we found evidence consistent with the hypothesis that the OVOLs regulate MET, but we also found evidence that the OVOLs might impact cancer in a broader sense. These results led us to make a three-way comparison of promoter occupancy across non-cancer, solid tumor, and leukemia models. The classic mechanism for metastasis of a solid tumor is EMT, migration, and MET [2]. This process is generally considered to be distinct from the mechanisms of progression in leukemia, though there are elements that are common across these cancer types [34,35]. To test these distinctions, we hypothesized that promoter occupancy would be higher in the solid tumor (MET) model than in the non-cancer model. Also, if the effect t is specific to the MET model, increased promoter occupancy would not be seen in the leukemia (Non-MET) model. If the effect is common to both MET and Non-MET mechanisms, we would see increased occupancy in both MET and Non-MET models, though the magnitude of the effect may be different.

We converted the downloaded the .csv ChIP-Seq files to .bed files and uploaded them to Genomatix GGA. We also converted the 4,102 promoter sequences from the OI-MET gene set to .bed files using the GGA mapping utility. For each TF, we aggregated the .bed files by tissue/cancer category (not cancer, MET, Non-MET). This process created proxy datasets for testing promoter occupancy, allowing us to look for documented binding of the TFs in sites overlapping the 4,102 promoters, in the relevant cellular models. For NFKB, only non-cancer ChIP-Seq data were available. For ATF3, only Non-MET data were available. For STAT1 and MYC, data were available for MET and Non-Met cancers, but not for the non-cancer model. For AP1, ChIP-Seq binding data were available for all three classes (not cancer, MET, and Non-MET cancers), for both FOS and JUN TFs. For all three tissues, for both FOS and JUN, we had relatively large numbers of peaks in each file to compare with the 4,102 promoters of interest, providing an excellent dataset for testing the hypotheses that these TFs occupy the promoters preferentially in MET versus non-cancer cells and in MET versus Non-MET cancer, but not in Non-MET versus non-cancer cells. Also, given that the AP1/MYC TF pair was the most enriched pair in the motif modules analysis, the AP1 ChIP-Seq data for FOS and JUN is a particularly good choice for this hypothesis testing. In addition, though we had only MET and Non-MET cancer data on MYC, we were able to test for enrichment of cooperative AP1/MYC binding in promoter associated locations in the cancer models.

### Overlap of ChIP-Seq AP1 binding peaks and promoter sequences

As seen in Table 7, we tested the overlap of AP1 ChIP-Seq peaks and the 4,102 promoters two ways (Tables 7 and 8), with each of JUN (upper) and FOS TFs (lower). In Table 7, upper sub-table (JUN, Promoter Occupancy), we considered the overlap of each promoter with at least one peak for the JUN TF. We tested the non-cancer set against the MET set, comparing the proportion of promoters overlapping non-cancer peaks (277) out of all non-cancer peaks (75,474), versus the proportion of promoters overlapping MET peaks (503) out of all MET peaks (120,679). We calculated fold change and p-value for this difference of proportions. We made the equivalent comparison but focused on the Non-MET peaks, relative to non-cancer peaks, then compared the MET peaks to the Non-Met peaks. The set of results in the lower sub-table (FOS) follow the same pattern as those in the upper sub-table, but FOS is the tested TF.

**Table 7:** **AP1** (**FOS and JUN**) **promoter occupancy in the OI**-**MET gene set**, **based on ChIPBase datasets**

	**Promoter occupancy (number of promoters overlapping with at least one peak)**			
**JUN**				
	**Not**_**cancer**	**MET**		
**Promoters with one or more peaks**	277	503		
**Peaks**	75474	120679	**Fold change**	**P-value**
**Promoter occupancy rate**	0.367%	0.417%	1.14	0.0966
	**not**_**cancer**	**Non**-**MET**		
**Promoters with one or more peaks**	277	483		
**Peaks**	75474	112929	**Fold change**	**P-value**
**Promoter occupancy rate**	0.367%	0.428%	1.17	0.0463*
	**MET**	**Non**-**MET**		
**Promoters with one or more peaks**	503	483		
**Peaks**	120679	112929	**Fold change**	**P-value**
**Promoter occupancy rate**	0.417%	0.428%	1.05	0.7083
**FOS**				
	**Not**_**cancer**	**MET**		
**Promoters with one or more peaks**	119	43		
**Peaks**	20695	3282	**Fold change**	**P-value**
**Promoter occupancy rate**	0.575%	1.310%	2.28	<0.0001**
	**Not**_**cancer**	**Non**-**MET**		
**Promoters with one or more peaks**	119	161		
**Peaks**	20695	37162	**Fold change**	**P-value**
**Promoter occupancy rate**	0.575%	0.433%	0.75	0.0226*
	**MET**	**Non**-**MET**		
**Promoters with one or more peaks**	43	161		
**Peaks**	3282	37162	**Fold change**	**P-value**
**Promoter occupancy rate**	1.310%	0.433%	0.33	<0.0001**

Promoter occupancy is slightly increased for JUN in both the MET and Non-MET models, relative to the non-cancer model, and there is essentially no difference in rates between the two models. Promoter occupancy is significantly increased for FOS in the MET model but is decreased in the Non-MET model, relative to the non-cancer model. The difference is highly significant. (*Significant at 0.05 P-value. **Significant at 0.001 P-value).

**Table 8:** **AP1** (**FOS and JUN**) **peak occupancy in the OI**-**MET gene set**, **based on ChIPBase datasets**

	**Peak occupancy (number of peaks overlapping with promoters)**			
**JUN**				
	**Not**_**cancer**	**MET**		
**Peaks overlapping promoters**	287	796		
**Peaks**	75474	120679	**Fold change**	**P-value**
**Peak occupancy rate**	0.38%	0.66%	1.73	<0.0001**
	**Not**_**cancer**	**Non-MET**		
**Peaks overlapping promoters**	287	757		
**Peaks**	75474	112929	**Fold change**	**P-value**
**Peak occupancy rate**	0.38%	0.67%	1.76	<0.0001**
	**MET**	**Non-MET**		
**Peaks overlapping promoters**	796	757		
**Peaks**	120679	112929	**Fold change**	**P-value**
**Peak occupancy rate**	0.66%	0.67%	1.02	0.7773
**FOS**				
	**Not**_**cancer**	**MET**		
**Peaks overlapping promoters**	103	34		
**Peaks**	20695	3282	**Fold Change**	**P-value**
**Peak occupancy rate**	0.50%	1.04%	2.08	0.0003**
	**Not**_**cancer**	**Non**-**MET**		
**Peaks overlapping promoters**	103	251		
**Peaks**	20695	37162	**Fold Change**	**P-value**
**Peak occupancy rate**	0.50%	0.68%	1.36	0.0105*
	**MET**	**Non**-**MET**		
**Peaks overlapping promoters**	34	251		
**Peaks**	3282	37162	**Fold Change**	**P-value**
**Peak occupancy rate**	1.04%	0.68%	0.65	0.0252*

Peak occupancy is significantly increased for both JUN and FOS, in both the MET and Non-MET models, relative to the non-cancer model. The effect is essentially the same for JUN in the MET and Non-MET models, but the effect for FOS is greater in the MET model. These results are consistent with both JUN and FOS transcription factors trans-locating to occupy the OI-MET promoters in both MET and non-MET models. (*Significant at 0.05 P-value. **Significant at 0.001 P-value).

Results in Table 7 (counting promoters overlapping one or more peaks) show that promoter occupancy is slightly increased for JUN in both the MET and Non-MET models, relative to the non-cancer model, and there is essentially no difference in rates between the two models. Promoter occupancy is significantly increased for FOS in the MET model but is decreased in the Non-MET model, relative to the non-cancer model. These results are strongly consistent with the hypothesis that FOS, as an element of the AP1 TF, impacts the MET model in the OI-MET gene set. The evidence for the Non-MET model is much less convincing.

Table 8 shows results for peak occupancy. The process for assessing enrichment is essentially the same as for promoter occupancy, but we are counting peaks that overlap promoters rather than counting promoters that overlap one or more peaks. These results are much more striking. In every case, there is a significant enrichment of peaks overlapping the OI-MET gene set’s promoters, for both JUN and FOS, for both the MET and Non-MET models. The effect of JUN is essentially the same in MET and Non-MET models. The effect of FOS is greater in the MET model, though we also see significant enrichment in the Non-MET model. These results are consistent with the hypothesis that both FOS and JUN, as elements of the AP1 dimer, impact the OI-MET gene set in both MET and Non-MET cancers. Taken together with results from Table 2, showing that FOS and JUN are responsive to the OVOLs, these results are consistent with the regulatory cascade described for Figure 10. In addition, the effect is not specific to the MET model.

### Enrichment of AP1/MYC peak pairs overlapping the OI-MET promoters

Based on the motif pair data, we hypothesized enrichment of binding by AP1/MYC pairs in our 4,102 promoters in the cancer models, relative to the non-cancer model. We tested this hypothesis (Table 9) in a way similar to how we tested for enrichment of AP1 binding in the cancer models. As in the previous analysis, Table 9 tests promoter occupancy and Table 10 tests peak occupancy. For both 8a and 8b, the upper sub-tables test JUN and the lower tables test FOS. In Table 9, for each model (MET, Non-MET) we first tested for the proportion of the 4,102 promoters occupied by only one member of the TF pair (e.g. Only JUN, Only MYC). Then, based on the proportion of promoters overlapping each single TF, and assuming that the TF binding sites are independent, we calculated the number of promoters that we would expect to have the TF pair (BOTH Expected). We then found the actual number of promoters overlapping both TFs (BOTH Observed). Using these observed and expected values we calculated Fold Change and p-value for the enrichment. In every comparison in Table 9, for both JUN and FOS matched with MYC, for both promoter occupancy and peak occupancy, we found very significant enrichment for overlap of both TFs with these promoters, in both MET and Non-MET cancer models. Also, we found a very large enrichment of peak occupancy (Table 10), relative to promoter occupancy (Table 9), in both cancer models. This result is consistent with the AP1/MYC pair having an important role in the cascade of gene expression regulation in the OI-MET gene set. Notably, AP1 was identified as being enriched in annotation in the OI-MET gene set, and MYC is the common target of OVOL1 and OVOL2, so this result is also consistent with the regulatory cascade described for Figure 10.

**Table 9:** **AP1** (**FOS and JUN**) ***with MYC *promoter occupancy in the OI**-**MET gene set**, **based on ChIPBase datasets**

**JUN**	**Promoter occupancy (# promoters overlapping both JUN and MYC peaks, from a total of 4,102 promoters)**					
	**MET**					
	**Only JUN**	**Only MYC**	**Both expected**	**Both observed**	**Fold change**	**P-value**
**Promoters with one or more peaks**	503	436	53.46	228	4.26	<0.0001**
	**Non-MET**					
	**Only JUN**	**Only MYC**	**Both expected**	**Both observed**	**Fold change**	**P-value**
**Promoters with one or more peaks**	483	398	46.86	288	6.15	<0.0001**
**FOS**	**Promoter occupancy (# promoters overlapping both FOS and MYC peaks, from a total of 4,102 promoters)**					
	**MET**					
	**Only FOS**	**Only MYC**	**Both expected**	**Both observed**	**Fold change**	**P-value**
**Promoters with one or more peaks**	43	436	4.57	22	4.81	<0.0001**
	**Non-MET**					
	**Only FOS**	**Only MYC**	**Both expected**	**Both observed**	**Fold change**	**P-value**
**Promoters with one or more peaks**	161	398	15.62	100	6.40	<0.0001**

Promoter occupancy is significantly enriched for both JUN and FOS with MYC, for both the MET and Non-MET models, relative to the expected if they were independent. This result is consistent with cooperative regulation of a subset of these promoters by AP1 and MYC. (**Significant at 0.0001 P-value).

**Table 10:** **AP1** (**FOS and JUN**) ***with MYC *peak occupancy in the OI**-**MET gene set**, **based on ChIPBase datasets**

**JUN**	**Peak occupancy (# of JUN and MYC peaks overlapping promoters, of 4,102 promoters)**					
	**MET**					
	**Only JUN**	**Only MYC**	**Both expected**	**Both observed**	**Fold change**	**P-value**
**Peaks overlapping promoters**	796	305	0.43	152	350.51	<0.0001**
**Peaks**	120679	19030				
	**Non-MET**					
	**Only JUN**	**Only MYC**	**Both expected**	**Both observed**	**Fold change**	**P-value**
**Peaks overlapping promoters**	757	910	0.31	670	2145.60	<0.0001**
**Peaks**	112929	80131				
**FOS**	**Peak occupancy (# of FOS and MYC peaks overlapping promoters, of 4,102 promoters)**					
	**MET**					
	**Only FOS**	**Only MYC**	**Both expected**	**Both observed**	**Fold change**	**P-value**
**Peaks overlapping promoters**	34	305	0.68	17	24.96	<0.0001**
**Peaks**	3282	19030				
	**Non-MET**					
	**Only FOS**	**Only MYC**	**Both expected**	**Both observed**	**Fold change**	**P-value**
**Peaks overlapping promoters**	251	910	0.31	253	804.10	<0.0001**
**Peaks**	37162	80131				

Peak occupancy is significantly enriched for both JUN and FOS with MYC, for both the MET and Non-MET models, relative to the expected if they were independent. This result is consistent with cooperative regulation of a subset of these promoters by AP1 and MYC. (**Significant at 0.0001 P-value).

## Discussion

In this work, we use a systems biology approach to understand how the OVOL TFs induce MET. Based on our previous work, we hypothesized that the OVOL TFs regulate MET in more than one cancer [3]. To test this hypothesis, we created models for OVOL Induced MET (OI-MET) in prostate cancer and breast cancer models, then found the common set of differentially expressed genes (the OI-MET signature). We used literature searches to test whether the OI-MET set is associated with appropriate terminology in PubMed and PMC and found significant evidence consistent with this hypothesis. Notably, this set is significantly associated with MET in the literature, as well as BC, PC, and cancer. We looked for the mechanisms by which the OVOL TFs regulate MET and found that only one third of the OI-MET genes promoters have the OVOL binding motif, so in most cases the mechanism is not likely to be direct OVOL TF binding. We then searched for other fundamental mechanisms acting in this set by enrichment testing with ConceptGen. We found significant enrichment for annotation consistent with cancer progression among genes in the OI-MET gene set, suggesting that the OI-MET set is a useful model of gene expression changes in MET.

We also found significant enrichment of annotation consistent with the roles of the OVOLs and AP1, NFKB1, STAT1, and STAT3 in regulating gene expression in OI-MET. To understand how these TFs might interact with the OVOL TFs and potentially impact OI-MET, we first focused the analysis on the subsets of genes associated with each of the five TFs (AP1, NFKB1, STAT1, STAT3, and the OVOL TFs), then assessed their interactions in the set composed of the union of the gene sets regulated by the individual TFs. This process yielded a model of how the OVOLs interact with the other TFs (OI-MET-TF) to influence OI-MET. We tested this model for association with BC, PC, cancer, and MET in the literature and found it to be even more enriched than the OI-MET model. This result is consistent with the hypothesis that the OI-MET-TF model is also useful in understanding the impact of the OVOLs in MET and more generally in cancer, as well as how the OVOLs interact with the other four TFs in this process. By developing an improved understanding the genes, interactions, and related mechanisms impacting disease, we open up the possibility of intervening in disease progression. We used the OI-MET-TF model to understand how known drug/gene interactions could impact the model and offer prioritized options for intervention.

We reflected our inference from the OI-MET-TF model back to the larger set of genes in the OI-MET signature and tested this gene set for potential regulation by these TFs. In the OI-MET gene set, we found significant enrichment for binding motifs for the AP1/MYC pair. To investigate potential binding at these sites, we used publicly available ChIP-Seq data to first test the hypothesis that AP1 binds preferentially in MET and Non-MET cancer models, relative to a non-cancer model. We also compared AP1 binding in the MET versus Non-MET models. Results of these tests are consistent with AP1 acting in both the MET and Non-MET cancer models. We then tested for preferential binding of the AP1/MYC pair, and again saw results consistent with this pair acting in both MET and Non-MET cancer models. While AP1 and MYC have long been associated with cancers, to our knowledge this is the first large scale test of the hypothesis that these TFs bind preferentially in cancer versus non-cancer models for cancer-related genes, and that they cooperate in binding. Taken together with evidence that FOS and JUN show differential expression in response to the OVOLs, these results are consistent with a regulatory cascade posed by the OI-MET-TF model. We also must consider the possibility that the OVOLs function in ways that are not specific to MET. This result has been seen with other transcription factors that were initially thought to act primarily in MET but were also found to impact cancer in ways not specific to MET [36].

## Conclusions

In this work, we explore the etiology of OVOL-Induced MET (OI-MET), focusing on commonalities between prostate cancer (PC) and breast cancer (BC) models, to test the hypothesis that the OVOLs regulate MET in multiple cancers. We generate a common OI-MET gene expression signature, consistent with a common underlying genetic etiology for MET in PC and BC, and show that the OI-MET gene set is significantly enriched for cancer, BC, PC, and MET associated genes. Using a systems biology approach, we identify regulation of gene expression as the primary influence of the OVOLs on MET, though this effect is indirect and depends on interaction with AP1, STAT1, STAT3, and NFKB1 TFs. We create an OI-MET-TF sub-model of the genes annotated as being regulated by the OVOLs and these other four TFs. We test this model for consistency with known genetic influences on MET, BC, PC and cancer, and find that there is significant evidence supporting the use of this network as a model of gene expression influences on MET. Based on these results, we believe the networks are useful in modeling the impact of the OVOLs and the four other TFs in MET, and may be appropriate for understanding broader influences in MET across multiple cancer types.

We use the OI-MET-TF model in several ways to improve our understanding of the mechanisms driving gene expression in MET. Based on the gene/drug and gene/gene interactions evident in the model, we prioritize known drugs for potential clinical application in cancer therapies. This analysis considers the potential for both on-target and off-target drug/gene interactions, as well as downstream effects and the possibility of repurposing drugs for novel cancer therapies. The OI-MET-TF model is also appropriate for future testing based on interactions with environmental factors, other risk genes, or potential drug therapies.

We extend the inference from the OI-MET-TF model back to the larger set of all OI-MET genes and show that the effects of the OVOLs and the other TFs in the OI-MET-TF model are likely to be consistent in the larger set, with experimental data significantly in support of this hypothesis. In particular, we find significant evidence that the AP1/MYC TF pair has an important role in regulating gene expression in cancers. In addition, we find that the impact of the OVOLs may extend beyond MET, influencing mechanisms of cancer progression that require further investigation.

## Methods

### RNA-seq sample preparation

The construction of PC and BC cell lines overexpressing OVOL1, OVOL2, or both was done as previously described [3]. Total RNA was isolated from biological replicates of each cell-type and subjected to deep transcriptome sequencing.

### RNA-seq data analysis

Sequencing was performed by the UM DNA sequencing core, using the Illumina Hi-Seq platform to generate 50 base, paired-end reads. We downloaded and concatenated the individual reads files to correspond with individual samples. These .fastq files are GEO datasets (GSE48230 and GSE51975). We aligned the reads to the reference transcriptome (UCSC hg19) using TopHat2.0.2, which is part of the tuxedo next-generation sequencing data analysis suite [37,38]. We used default parameter settings with the exception that we specified “–b2-very-sensitive”. We used FastQC (http://www.bioinformatics.babraham.ac.uk/projects/fastqc/) to assess a range of quality measures, and found overall very good quality aligned reads in each sample. We then used CuffDiff2.0.2, also part of the tuxedo suite, to assess differential expression between groups, using the UCSC hg19.gtf transcriptome, with -u, −N, −-compatible-hits-norm, and -b (relative to the UCSC hg19.fa) parameter settings. We used a locally derived Perl script to identify genes/transcripts as being differentially expressed if they showed: “OK” test status AND FDR ≤ 0.05, AND fold change (≥ 2.0 or ≤ 0.50).

### E-Utils publications searches

We used Perl script with NCBI Entrez­ Programming Utility functions (e-utilities) to query NCBI literature databases. For each gene in the list, we queried both PubMed and PubMed Central databases using HGNC gene symbol and each of the terms “cancer”, “breast cancer” and “prostate cancer” as text words and “epithelial mesenchymal transition” as the MeSH term for MET. Each query result was parsed to get a list of PMIDs and PMCIDs, respectively, that document the co-occurrence of the gene symbol and the query term. We counted co-occurrence if one or more publication showed both the gene symbol and the query term.

### GeneGo network building

For each network, we used parameter settings to develop the most parsimonious network possible (including all the input genes in the smallest possible network). In each case, we used: shortest paths algorithm; merged network; not including canonical pathways; 1 maximum step in the path; showing disconnected seed nodes; showing shortest path edges only; using low trust, functional, and binding interactions for network building, and not using compound-target interactions. Note that, while we allowed for the potential use of low trust interactions in network building, this did not impact the networks built.

## Abbreviations

MET: Mesenchymal-epithelial transition; OI-MET: Ovol-induced MET; TF: Transcription factor; OI-MET-TF: OI-MET transcription factor model.

## Competing interests

KJP is a member of the board of Curis, Inc., and Oncofusion Therapeutics, Inc.

## Authors’ contributions

RCM, HR, KJP, and JDC conceived the project and developed an outline for the work. MP developed the E-Utils scripts and performed multiple rounds of analysis to develop the data for hypothesis testing. JSH assessed the gene/drug interactions and potential clinical implications of the OI-MET-TF model. JH and HR developed the samples for input to the sequencing core for RNA-Seq analysis. HR, KJP, and JDC provided context for the biological implications of the work. RCM and HR performed the RNA-Seq data analysis. RCM generated the networks, performed hypothesis testing, and generated the initial draft of the manuscript. All authors edited the manuscript and approved the final draft.

## Supplementary Material

Additional file 1 **Differentially expressed genes in the OI-MET gene set.** Excel spread sheet with set of genes at the intersection of BC and PC models, yielding the OI-MET gene set. Click here for file

Additional file 2 **ConceptGen enrichment testing results for the OI-MET gene set.** Excel spread sheet with results from the ConceptGen enrichment testing, sorted by category, with the five TF concepts at the top of the page. Click here for file

Additional file 3 Metacore quick reference guide. Click here for file

Additional file 4 **GeneGo MetaDrug gene/drug interactions.** Excel spread sheet with connections between network genes, their descriptions, and the therapeutic drugs that target them. Click here for file
