# Supplementary material for: A bioinformatics approach reveals novel interactions of the OVOL transcription factors in the regulation of epithelial – mesenchymal cell reprogramming and cancer progression
Source: BMC Syst Biol. 2014 Mar 10;8:29. doi: 10.1186/1752-0509-8-29 (PMC4008156; doi:10.1186/1752-0509-8-29)
Supplement: Additional file 3 — Metacore quick reference guide. [file 1752-0509-8-29-S3.pdf]

METACORE QUICK REFERENCE GUIDE

USER DATA

NETWORKS / MAPS

Up-regulated (+)
Object has user data with positive value

Down-regulated (-)
Object has user data with negative value

Mixed-signal (+/-)
Object has user data with both positive and negative values

Gene variants
Object has user data with gene variants

Mixed data
Object has user data with both expression values and gene variants

NETWORK OBJECTS

ENZYMES

Generic enzyme

KINASE
- Generic kinase
- Protein kinase
- Lipid kinase

PHOSPHATASE
- Generic phosphatase
- Protein phosphatase
- Lipid phosphatase

PHOSPHOLIPASE
- Generic phospholipase

PROTEASE
- Generic protease
- Metalloprotease

GTPASE
- G-alpha
- RAS - superfamily

GENERIC CLASSES

- Receptor ligand
- Transcription factor
- Protein
- Compound
- Predicted metabolite or user's structure
- Inorganic ion
- Reaction
- DNA
- RNA
- Generic binding protein

CHANNELS/TRANSPORTERS

- Generic channel
- Ligand-gated ion channel
- Voltage-gated ion channel
- Transporter

RECEPTORS

- Generic
- GPCR
- Receptors with kinase activity

G PROTEIN ADAPTOR/REGULATORS

- G beta/gamma
- Regulators (GDI, GAP, GEF, etc.)

GROUPS OF OBJECTS

A complex or a group
Proteins physically connected into a complex or related as a family

Logical association
Proteins linked by logical relations or physical interactions

Custom association
Group of collapsed objects chosen by user

Group1

INTERACTIONS BETWEEN OBJECTS

EFFECTS

- Positive / activation
- Negative / inhibition
- Unspecified

MECHANISMS

PHYSICAL INTERACTIONS

B — Binding
Physical interaction between molecules

C — Cleavage
Cleavage of a protein at a specific site yielding distinctive peptide fragments. Proteolytic cleavage can be carried out by both enzymes and compounds

CM — Covalent modifications
Covalent binding of a small chemical groups to protein amino acids or DNA/RNA nucleotides.

+P — Phosphorylation
Protein activity is altered via addition of a phosphate group

- P — Dephosphorylation
Protein activity is altered via removal of a phosphate group

T — Transformation
Protein activity regulation by binding & hydrolysis of GTP

Tn — Transport
Transport of a protein or a compound between organelles

Z — Catalysis
Catalysis of an enzymatic reaction

Tr — Transcription regulation
Physical binding of a transcription factor to target gene's promoter

cRT — Co-regulation of transcription
Influences on gene expression by direct binding with transcription machinery or by chromatin remodelling

Rg — Regulation
Influence on the biochemical reaction by changing its composition

M — MicroRNA binding
Regulation of gene expression by binding of microRNA to target mRNA

FUNCTIONAL INTERACTIONS

IE — Influence on expression
indirect influence of chemical compound or protein on the amount of another protein

Cn — Competition
When two molecules compete for the interaction with the third molecule

? — Unspecified interactions
Influence on activity of protein or RNA without determined mechanism

Pr — Processing
Protein is a product of posttranslational modification.

PE — Drug-Drug interactions. Pharmacological effect
Drugs change pharmacological effects of other drugs, for instance by competing for drug metabolism enzymes or organic transporters

TE — Drug-Drug interactions. Toxic effect
Drugs change toxic effects of other drugs, for instance by competing for drug metabolism enzymes or organic transporters

LOGICAL RELATIONS

GR — Group relation
Object belongs to a generic group of related objects

CS — Complex subunit
Protein is a subunit of a protein complex

SR — Similarity relation
Chemically similar compounds with chosen Tanimoto similarity score

LINKS ON NETWORKS

Incoming interaction
When the mouse is over object, yellow link indicates direction to object

Outgoing interaction
Cyan link indicates direction FROM the object

INTERACTIONS FROM CUSTOM LIST (MetaLink™)

Interaction is in the network
Interaction is represented by a thin solid line and is highlighted in blue

Interaction is in the base, but not in network
Interaction is highlighted in yellow

Interaction is in the network
Interaction is highlighted in magenta

CANONICAL PATHWAYS

Canonical pathway
The link is highlighted in a thick cyan or magenta line

LINKS ON MAPS

- Disrupts in disease
- Weakens in disease
- Emerges in disease
- Enhances in disease
- Species specific interactions

OBJECTS ON MAPS

LOCALIZATION

- Mitochondria
- EPR
- Golgi
- Nucleus
- Lysosome
- Peroxisome
- Cytoplasm
- Extracellular

OTHER MAP OBJECTS

Comments
- Note
- Normal process
- Pathological process

Blocks
- Normal process
- Pathological process

- Species specific object
- Path start

THOMSON REUTERS REGIONAL OFFICES

North America
Philadelphia
+1 800 336 4474
+1 215 386 0100

Latin America
+55 11 8370 9845

Europe, Middle East and Africa
Barcelona +34 93 459 2220
London +44 20 7433 4000

Asia Pacific
Singapore +65 6775 5088
Tokyo +81 3 5218 6500

Contact us to find out more about *MetaCore* or visit **thomsonreuters.com/diseaseinsight**

For a complete office list visit: **science.thomsonreuters.com/contact**

LS0312

Copyright © 2013 Thomson Reuters

THOMSON REUTERS™
